# Recurrent Laryngeal Nerve Liberations and Reconstructions: A Single Institution Experience

**DOI:** 10.1007/s00268-015-3305-0

**Published:** 2015-11-09

**Authors:** Radan Dzodic, Ivan Markovic, Nada Santrac, Marko Buta, Igor Djurisic, Silvana Lukic

**Affiliations:** School of Medicine, University of Belgrade, Belgrade, Serbia; Surgical Oncology Clinic, Institute for Oncology and Radiology of Serbia, Pasterova 14, 11000 Belgrade, Serbia; Department of Pathology, Institute for Oncology and Radiology of Serbia, Belgrade, Serbia

## Abstract

**Background:**

Recurrent laryngeal nerve (RLN) palsy rates vary from 0.5 to 10 %, even 20 % in thyroid cancer surgery. The aim of this paper was to present our experience with RLN liberations and reconstructions after various mechanisms of injury.

**Methods:**

Patients were treated in our institution from year 2000 to 2015. First group (27 patients) had large benign goiters, locally advanced thyroid/parathyroid carcinomas, or incomplete previous surgery of malignant thyroid disease. Second group (5 patients) had reoperations due to RLN paralysis on laryngoscopy. Liberations and reconstructions of injured RLNs were performed.

**Results:**

Surgical exploration of central compartment enabled identification of the RLN injury mechanism. Liberations were performed in 11 patients, 2 months to 16 years after RLN injury, by removing misplaced ligations. Immediate or delayed (18 months to 23 years) RLN reconstructions were performed in 21 patients, by direct suture or ansa cervicalis-to-RLN anastomosis (ARA). RLN liberation provided complete voice recovery within 3 weeks in all patients. Patients with direct sutures had better phonation 1 month after reconstruction. Improved phonation was observed 2–6 months after ARA in 43 % of patients.

**Conclusions:**

Vocal cords do not regain normal movement once being paralyzed after RLN transection, but they restore tension during phonation by reconstruction. Nerve liberation is a useful method which enables patients with RLN paresis/paralysis a significant improvement in phonation, even complete voice recovery. Reinnervation of vocal cords, using one of the mentioned techniques, should be a standard in thyroid and parathyroid surgery, with aim to improve quality of patient’s life.

## Introduction

Thyroid surgery is the surgery of the laryngeal nerves and parathyroid glands. Recurrent laryngeal nerve (RLN) palsy reported rates vary in the relevant literature from 0.5 to 10 % [1–7]. Incidence of RLN injuries increases with the extent of surgery and is higher in thyroid cancer surgery with extensive dissections, reaching up to 20 % [7, 8].

Visual identification and meticulous dissection of RLN presents a “gold standard” in thyroid surgery. Extrathyroid tumor extension and lymph node metastases along RLNs interfere with RLN identification and adequate preservation. Intraoperative nerve monitoring (IONM) has been proposed as an adjunct to visual identification [9–11]. However, RLN injuries were recorded using IONM, as well [12, 13].

Pathoanatomic settings for iatrogenic RLN injuries are various (adhesions in thyroiditis, large hypervascular goiters, previous surgery, or radiation). Injury mechanisms include traction, contusion, thermal damage, misplaced ligation, and complete or partial transection. Lack of surgeon’s experience is recognized as a significant risk factor for RLN palsies, as well [12].

Quality of life in patients with RLN palsy, especially permanent, is severely reduced. Several surgical methods can provide voice improvement for these patients, but only to a certain extent, depending on the injury type and the surgeon’s skills. The first author of this paper (RD), with his surgical team, performs yearly around 700 operations on thyroid and parathyroid glands. The aim was to present experience with liberations and reconstructions of RLNs after various mechanisms of injury and to discuss the recovery of patients.

## Materials and methods

From year 2000 to 2015, at the Surgical Oncology Clinic of the Institute for Oncology and Radiology of Serbia, our surgical team performed RLN liberations and reconstructions in 32 patients during operations and reoperations due to different indications. These included large benign goiters (toxic and non-toxic), recurrent goiters, locally advanced thyroid/parathyroid carcinomas, or incomplete thyroid cancer surgery performed previously in other hospitals. Permanent, symptomatic RLN paralysis, in patients previously treated outside our institution, has been distinguished as a special indication for reoperation and RLN reconstruction, with aim to improve quality of patient’s life.

### Treatment protocol

Patients were preoperatively presented to the multidisciplinary committee at our Institute. Diagnostic procedures were the following: neck ultrasonography, thyroid hormone status, laryngoscopy, chest X-ray, and abdominal ultrasonography in all patients, and neck and chest MRI/CT scan, bronchoscopy, esophagoscopy, whole body scintigraphy, when indicated. Written consent was obtained for the procedure and the possible complications of the surgery.

### Surgical techniques for RLN reparation

Surgical approach was through standard neck skin incision for thyroidectomy. Exploration of thyroid bed and central neck compartment was performed through median approach or via “back door” in reoperations. After visually identifying the RLN and the injury mechanisms, liberations or reconstructions were performed.

#### RLN liberation technique

As a part of reoperations for above-listed indications, visual identification of RLN was achieved via “back door” approach and sharp dissection. Misplaced ligation on the intact RLN was observed adjacent to neurovascular crossing of the RLN and the inferior laryngeal artery. By meticulous removing of the ligation, RLN was deliberated and its integrity was visually confirmed. In two patients, granulomas formed around surgical suture near the RLN compressed the RLN at the laryngeal entry point, and thus, these were sharply removed. This technique of RLN liberation was originally presented by the first author (RD), as well as published in the form of abstract [14]).

#### Direct, “end-to-end” RLN anastomosis

In primary surgeries, after transection of RLN, immediate reconstruction was performed by a direct, “end-to-end” anastomosis of neural stamps, by three to four perineural stitches of 7-0 nylon thread, using microsurgical instruments. In reoperations, after transection due to severe adhesions or after “back door” identification of previously transected RLN, distal and proximal stumps were sutured in the same way.

#### Ansa cervicalis to RLN anastomosis (ARA, Miyauchi’s technique [15, 16])

First, we identified ansa cervicalis on the surface of the internal jugular vein, and branches to the sternothyroid muscles were dissected. The proximal end of the major branch was anastomosed to the distal RLN stump. Ipsilateral ansa cervicalis was used for reinnervation in all patients but one, in whom it was resected during previous lateral neck dissection performed in other hospital. Here, contralateral ansa cervicalis was used.

### Treatment outcome evaluation

The voice quality and other disease-related symptoms and signs were routinely noted in the medical chart of every patient, prior to operation and later on postoperative check-ups, prospectively. For evaluation of the outcome of RLN liberation and reconstruction techniques, all symptoms and signs of RLN palsy were used for defining a qualitative scale with scores from 0 to 5:

0—stridorous breathing, severe dysphonia, vocal fatigue, whisper voice;

1—stridorous breathing on exertion, dysphonia, vocal fatigue, whisper voice;

2—no stridorous breathing, mild dysphonia, vocal fatigue, variations in voice tone;

3—better phonation, occasional dysphonia, occasional vocal fatigue, variations in voice tone;

4—good phonation, no vocal fatigue; and

5—normal phonation.

Described scoring system is an unvalidated scoring system, created by the authors for the purpose of our daily clinical practice.

Other than this perceptual voice quality assessment, vocal cords position and movements were recorded prospectively by laryngoscopy examinations in the 1st, 6th, and 12th month after operation. All patients had phoniatric rehabilitation and are still in the follow-up.

## Results

### Pathology findings

In the reported series of 32 patients, 22 had papillary thyroid carcinoma (PTC), two had medullary thyroid carcinoma (MTC), one had parathyroid carcinoma, six patients had benign toxic or non-toxic goiter, and one follicular adenoma. All carcinoma patients had T4aN1a/b tumors [17].

### Indications, surgical treatment, and RLN injury mechanisms

In the first group of 27 patients, indications for surgery were large benign goiters, locally advanced thyroid/parathyroid carcinomas, recurrent goiters, or incomplete thyroid cancer surgery performed previously in other hospitals.

**Fig. 1:**
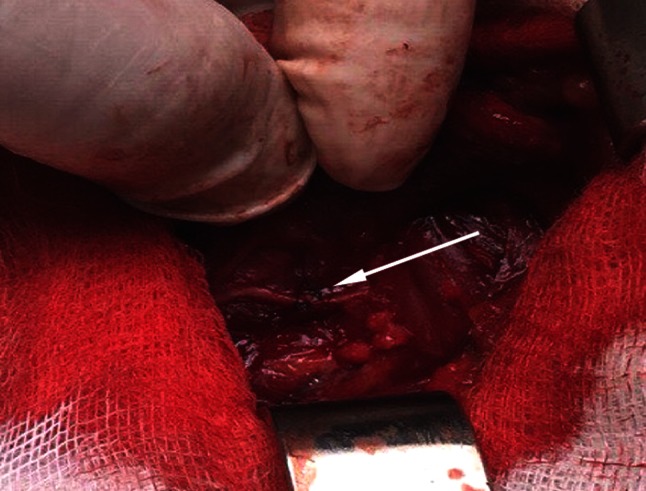
Intraoperative photo of accidentally transected left recurrent laryngeal nerve, immediately reconstructed by direct anastomosis of transected neural ends using 7-0 nylon thread perineural sutures (*white arrow*). Photo was originally taken during one of the primary surgeries due to a large follicular lesion of the left thyroid lobe, performed by first author (RD) and his team in our institution
- Reoperations were performed in 15 patients (Table 2). Extensive lymph node dissections and central neck compartment re-dissections had to be done in 13 patients due to incomplete surgery of malignant disease (PTC in 12, MTC in one case). In the remaining two cases, completion thyroidectomy was performed due to recurrent benign goiter. Eleven patients were preoperatively diagnosed on laryngoscopy for having RLN paralysis (7 patients) or paresis (4 patients). Intraoperative exploration revealed misplaced ligation on RLN in nine patients, while two had granulomas near the RLN laryngeal entry point, causing nerve paralysis. During reoperation, RLN liberation was performed by removing misplaced ligations (Fig. 2) and granulomas. In four out of 15 patients, adequate visual identification of RLN was hardly possible due to extensive postoperative adhesions and severe distortion of anatomic landmarks, which led to RLN transection. Immediate reconstruction was done: in three patients by ARA (Fig. 3) and in one by direct anastomosis.

- Primary surgery was performed in 12 patients (Table 1). In three of these, accidental unilateral RLN transection injuries were recorded due to significant distortion of anatomic landmarks in large, multinodular goiters or large follicular lesion. These injuries were treated with immediate reconstruction by direct anastomosis (Fig. 1). Remaining nine patients had unilateral malignant tumor infiltration of the RLN, or large lymph node metastases along RLN, infiltrating the nerve. Tumors were mostly PTCs, but isolated cases of MTC and parathyroid carcinoma were observed, as well. RLN infiltration was previously suspected of in two patients with RLN paralysis on preoperative laryngoscopy and significant dysphonia. Other seven patients had no vocal cord movement disorder, but moderate dysphonic problems were noted. These cases with RLN infiltration could not be managed by partial resection of the neural layer, and required a RLN transection in order to achieve “clear” resection margins. Immediate reconstruction by direct anastomosis was possible in one patient, while ARA technique was applied in other eight patients.

**Table 1:** Recurrent laryngeal nerve injury mechanisms during primary thyroid and parathyroid surgery with techniques of repair

Injury mechanism	*N* ^a^	Pathology		Preoperative laryngoscopy	Repair time	Repair mechanism	
		Benign	Malignant			DA^b^	ARA^c^
Accidental transection	3	3	0	normal	Immediate	3	0
Nerve resection due to infiltration	9	0	9	Paralysis (2) normal (7)	Immediate	1	8
Total	12	3	9	/	/	4	8

^a^Number of patients

^B^Direct anastomosis

^c^Ansa cervicalis to recurrent laryngeal nerve anastomosis

**Table 2:** Recurrent laryngeal nerve injury mechanisms verified during reoperative thyroid surgery with techniques of repair

Injury mechanism	*N* ^a^	Pathology		Injury time	Preoperative laryngoscopy	Repair time	Repair mechanism		
		Benign	Malignant				DA^b^	ARA^c^	L^d^
Accidental transection	4	0	4	Secondary surgery	Normal	Immediate	1	3	0
Misplaced ligation	9	1	8	Initial surgery	Paralysis (5) or paresis (4)	Delayed	0	0	9
Granulomas	2	1	1	Initial surgery	Paralysis	Delayed	0	0	2
Total	15	2	13	/	/	/	1	3	11

^a^Number of patients

^b^Direct anastomosis

^c^Ansa cervicalis to recurrent laryngeal nerve anastomosis

^d^Liberation

**Fig. 2:**
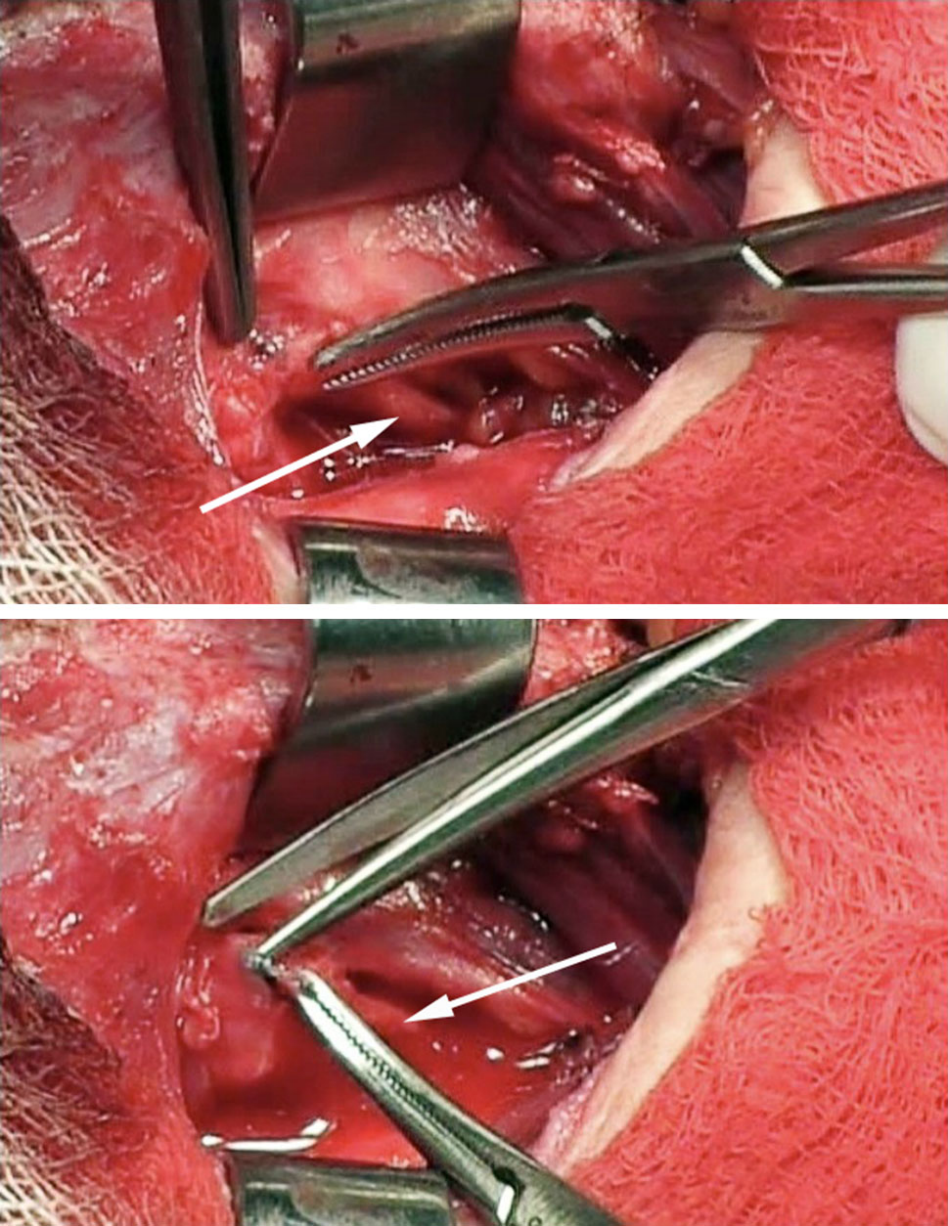
Intraoperative photos showing recurrent laryngeal nerve liberation technique. On the upper photo, tip of the tweezer is showing a misplaced ligation on the right recurrent laryngeal nerve (pointed by *white arrow*), at its laryngeal entry point. On the lower photo, misplaced ligation is retracted by the Mosquito forceps, while the ligation is being meticulously removed by scissors. Photos were originally taken during one of the reoperations of thyroid carcinoma, performed by the first author (RD) and his team in our institution

**Fig. 3:**
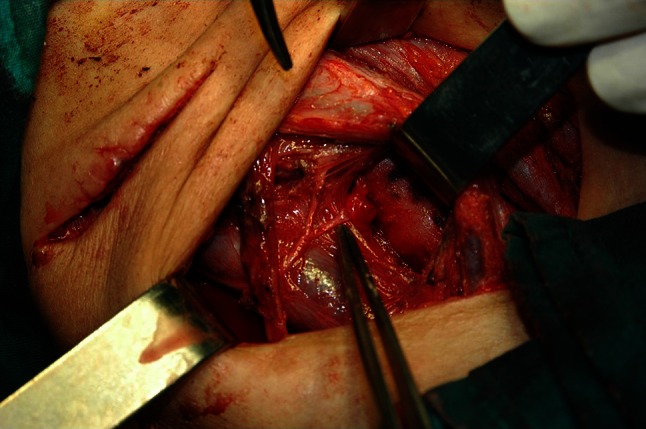
Intraoperative photo showing ansa cervicalis to recurrent laryngeal nerve (RLN) anastomosis (ARA) on the right side. The right ansa cervicalis neural fiber used for reconstruction is crossing over the right internal jugular vein and is pointed by the tip of the tweezer. It is anastomosed with distal stump of the right RLN at the laryngeal entry point, using 7-0 nylon thread perineural sutures. RLN was infiltrated by papillary thyroid carcinoma (pT4aN1b), without possibility for partial layer resection of the nerve. In order to achieve “clear” resection margins, RLN was transected, and immediately reconstructed by ARA technique. Total thyroidectomy, complete central dissection, and right modified radical neck dissection on two incisions were performed by the first author (RD) and his team during primary surgery in our institution

In the second group of 5 patients (Table 3), permanent, symptomatic RLN paralysis, after initial PTC surgeries performed in other hospitals, was the only indication for reoperative surgery. Patients had severe dysphonic problems, hoarseness, vocal fatigue, whisper voice, and stridorous breathing. RLN paralysis was confirmed by laryngoscopy examination before the reoperation and neurapraxia was excluded based on time lapsed from injury (over 12 months). One patient had bilateral RLN paralysis, two patients had RLN paralysis on one side and paresis on the opposite side, and the remaining two were with unilateral RLN paralysis on laryngoscopy. In two patients, direct anastomosis was possible, while in other three ARA was done. Surgical exploration with RLN reparation was unilateral in all but one patient, who received bilateral repair of injured RLNs (direct anastomosis and liberation) in two consecutive operations.

**Table 3:** Group of patients with severe symptomatology and uni/bilateral recurrent laryngeal nerve paralysis on preoperative laryngoscopy, that were reoperated for a delayed repair of the injured recurrent laryngeal nerve, with preoperative (initial) and postoperative (final) scores

	Pathology	RLN injury	Injury mechanism	Preoperative laryngoscopy	Time to repair^c^	Repair mechanism	Initial score	Final score
Patient No. 1	PTC^a^	Unilateral	Transection	Paralysis	23years	DA^d^	2	4
Patient No. 2	PTC^a^	Bilateral	Transection	Paralysis	24 m	DA^d^	1	3
			Misplaced ligation	Paresis	27 m	L^e^		
Patient No. 3	PTC^a^	Bilateral	Transection	Paralysis	18 m	ARA^f^	0	3
			Unknown^b^	Paralysis	/	/		
Patient No. 4	PTC^a^	Bilateral	Transection	Paralysis	24 m	ARA^f^	1	3
			Unknown^b^	Paresis	/	/		
Patient No. 5	PTC^a^	Unilateral	Transection	Paralysis	60 m	ARA^f^	2	3

^a^Papillary thyroid carcinoma

^b^Surgical exploration was unilateral, only one recurrent laryngeal nerve was reconstructed with ARA, while the injury mechanism of the opposite paretic/paralytic recurrent laryngeal nerve is unknown

^c^Time from initial operation, i.e., nerve injury, to recurrent laryngeal nerve repair

^d^Direct anastomosis

^e^Liberation

^f^Ansa cervicalis to recurrent laryngeal nerve anastomosis

### Liberations of RLNs and outcome

Liberations were performed in 11 patients, 2 months to 16 years after RLN injury that occurred during initial surgery in other hospital (Table 2). Nine patients were reoperated due to incomplete previous surgery of PTC, one due to recurrence of Graves’ disease, and one due to recurrent, symptomatic non-toxic goiter. All patients had RLN palsy on preoperative laryngoscopy examination (paralysis in 7, paresis in 4 patients) and mild to severe dysphonic symptoms, as well as stridorous breathing in bilateral injuries. During reoperation, surgical exploration of the RLNs revealed misplaced ligations and granulomas in nine and two patients (respectively), which were further removed sharply. Two patients had bilateral RLN paralysis due to misplaced ligations on both RLNs during initial PTC and Graves’ disease surgeries (respectively). Liberations in these two cases were performed in two-step surgery: 2 and 6 months after injury. RLN liberations provided complete voice recovery within three weeks in all patients (Table 4). In 10 patients, score 4 on perceptual voice quality scale was achieved. One patient, who had RLN liberation 16 years after the injury, restored normal vocal cord movements on laryngoscopy, with score 5 on voice quality scale.

**Table 4:** Outcome of patients after recurrent laryngeal nerve repair by different surgical techniques, assessed by a qualitative scale

Repair mechanism	Time of evaluation (after repair)	Number of patients					
		Score 1	Score 2	Score 3	Score 4	Score 5	Total
Liberation	3 weeks	/	/	/	10	1	11
Immediate DA^a^	6 months	/	/	/	5	/	5
Delayed DA^a^	6 months	/	/	1^c^	1	/	2
Immediate ARA^b^	12 months	/	/	11	/	/	11
Delayed ARA^b^	12 months	/	/	3	/	/	3
Total	/	/	/	15	16	1	32

^a^Direct anastomosis

^b^Ansa cervicalis to recurrent laryngeal nerve anastomosis

^c^Patient with bilateral recurrent laryngeal nerve injury, who received a delayed DA of one and deliberation of the other paretic recurrent laryngeal nerve

### Reconstructions of RLNs and outcome

RLN reconstructions were performed in 21 patients: immediate in 16 patients, while delayed in 5–18 months to 23 years after RLN injury in other hospital.

Direct anastomosis was performed in seven patients: in five immediately after injury, while in two patients as a delayed procedure (2 and 23 years after injury). All patients with immediate reconstruction had better phonation one month after the procedure, with occasional vocal fatigue (score 3). Six months later (Table 4), phonation was good, without fatigue (score 4), although mobility of vocal cord could not be achieved. Individual outcomes of patients with delayed direct anastomosis are given in Table 3. One patient, given a 23-year-delayed reconstruction by direct anastomosis, had complete loss of dysphonic symptoms (score 4) and small amplitudes of vocal cord movement were verified on laryngoscopy.

ARA was performed in 14 patients: in 11 immediately, while in three patients as a delayed procedure (18, 24, and 60 months after injury). Improved phonation (score 3) was observed 2–6 months after ARA in six (43 %) patients with immediate reconstruction. In 12th postoperative month (Table 4), all patients with ARA, both immediate and delayed, achieved score 3 (100 %). However, no vocal cords movement was noted on laryngoscopy. Individual outcomes of patients with delayed ARA are given in Table 3.

## Discussion

Expected RLN position in the neck can be significantly changed, especially in large goiters and gross central lymph node metastases, which can lead to RLN injury even in highly experienced surgical teams. Extralaryngeal terminal bifurcation of RLNs is an anatomic setting with risk for RLN injuries [18]. Patients undergoing thyroid surgery for malignancies and reoperations have increased risk of RLN injury [8]. Morbidity is higher in hands of inexperienced surgeons [12, 15].

Mechanisms of RLN injuries are various. Some, like traction, contusion, or thermal damage, are not evident intraoperatively due to macroscopically preserved integrity of the nerve, but can be verified using IONM. Consequent neurapraxia is a reversible condition, with complete vocal cords movement recovery within 12 months from injury. Accidental RLN ligation sometimes can be visually missed, but it will certainly be verified during the reoperation by an experienced surgeon. In malignant infiltration of RLN, when shave-off is impossible, or “clear” resection margins cannot be achieved by partial layer resection, RLN transection is inevitable and must be treated by immediate reconstruction [19].

The first direct RLN anastomosis was described by Horsley in 1910 [20]. Crumley and Izdebski first reported ARA in selected patients with unilateral vocal cord paralysis in 1986 and 1991 [21, 22], and few years later Miyauchi [23, 24].

Not many authors in the relevant literature report reconstructions of RLNs with successful outcome. However, they all agree upon urgency of surgical exploration and prompt reconstruction of the RLN [25–30]. Miyauchi et al. report the longest period of vocal cord paralysis of 4 and 6 years [25]. Here, we report a delayed reconstruction by direct anastomosis 23 years after the RLN injury in one patient, with full recovery regarding dysphonic symptoms and small amplitudes of vocal cord movements on laryngoscopy. This implies that not only prompt, but a late surgical exploration and reconstruction, as well, can be useful and result in better quality of life for a patient with RLN injury. Certainly, time from the appearance of dysphonic symptoms to decision upon the reoperation for RLN repair should not be less than 12 months, as there is the possibility of RLN recovery from neurapraxia.

Literature review on RLN reconstructions offers the term “*decompression*” that covers a wide range of surgical techniques and procedures, including RLN reparation. Successful decompressions were reported in early and mid-stage surgical exploration [31, 32]. We report a complete voice recovery in one patient with RLN paralysis in whom RLN liberation was performed 16 years after the injury. On postoperative laryngoscopy, patient had normal vocal cord position and movements, with normal phonation.

Maximum phonation time and phonation efficiency index, as methods for quantitative evaluation of voice recovery [25], are not used in our patients due to technical inability of our institution. Instead, the authors have used a perceptual voice quality assessment and clearly showed voice recovery after liberations and both immediate and delayed reconstructions.

Nerve reconstruction is a procedure that depends on surgeon’s skills and experience. Although vocal cords do not regain normal movement once being paralyzed, they can restore tension during phonation by reconstruction [12, 33]. Liberation of ligated RLNs is a very comfortable procedure that provides complete voice recovery within a few weeks from operation. It is a useful method which enables patients with RLN paresis/paralysis a significant improvement of phonation, and should be indicated in all cases when misplaced ligation is verified intraoperatively. It is especially beneficial for patients with severe symptomatology and poor quality of life. There are other established methods of voice improvement, as well, such as laryngoplasty [34–36]. However, in our institution, we only have experience with RLN reconstructions and liberations.

The authors find these results encouraging for introducing the new indication for reoperations—a permanent, symptomatic RLN paralysis. RLN repair is possible even years after the injury. Reinnervation of vocal cords, using one of the mentioned techniques, should be a standard method in thyroid and parathyroid surgery, with aim to improve patients’ quality of life.
